# Therapeutique Appliquée, ou Traitements Speciaux de la plupart des Maladies Chroniques

**Published:** 1844-10-01

**Authors:** 


### THERAPEUTIQUE APPLIQUÉE, OU TRAITEMENTS SPECIAUX DE LA PLUPART DES MALADIES CHRONIQUES. Par *P. J. C. Debreyne.* 2me. Edition, pp. 332. Baillière, 1844.

THIS is really a very useful and instructive work. It contains the results of our author's experience, over a period of nearly thirty years, in a variety of those chronic diseases which are of most frequent occurrence. Dr. *Debreyne* is evidently a shrewd and practical observer; he has learned to think and reason for himself; and seems to have had, throughout his professional life, a marked aversion for all the nosological theories which have occupied so largely the attention of most of his countrymen during the present century. See, how he treats the chiefs of philosophical (!) medicine:

"The Pinel-ists, the organicians, the anatomo-pathologists, the Broussais-ians, the statisticians, the numerists have, all, by the exclusiveness of their particular doctrines and views, stood in the way of, and materially retarded the advance of sound therapeutic knowledge. Now, however, that the system of universal irritation and of a materialist physiologism has fallen to pieces, a new era has happily opened up to our view, and Hippocratic vitalism has re-appeared amongst us in all its primitive splendour. . . . . The reign of anatomism, that is to say of necropsies and facts and figures, has nearly come to an end; and medical men now long for something more tangible and more applicable to the every-day duties of a professional life; in other words, they wish to have pointed out to them useful rules of treatment and rational means of cure, instead of endless catalogues of statistic tables and of post-mortem examinations."

Dr. D. is professor of practical medicine to the establishment of Grande-Trappe (Orne), and seems to have reared a number of pupils, who have contributed, for some years past, not a little to disseminate his peculiar doctrines and modes of treatment in different districts of France. He is moreover, the author of several treatises—on Physiology, Hygiene, Moral Theology in its relations with Medicine, of which we gave a short notice in the last number of this Review. However much we may feel inclined to dissent from him on several points of practice, we have been decidedly pleased with the general tone of the present work, which appears to be a faithful record of discriminating observation of disease at the bed-side of his patients. There is nowhere any parade of learned phrases ; no darkening of knowledge with a multitude of words; no wearisome and most profitless description of very common cases ; no heaping together of other men's opinions and doings, with a hesitating announcement of his own. Instead of this, we have a plain unvarnished tale of what the author has seen and found in practice; and all this explained in as few words as possible. In fine, this book is thoroughly and essentially a practical one —a somewhat uncommon feature, by the bye, of a French medical work in the present day. Its motto is *experire*: our readers cannot do better than accept the challenge and judge for themselves.

The diseases, which pass under review, are arranged in three divisions —Neuroses or Neuropathies; Chronic Phlegmasiæ ; and Astheniæ. We begin with a short notice of

*Epilepsy*.—The remedy, which Dr. D. has found by far the most successful in the treatment of this disease when it is idiopathic, and there are no symptoms of existing cerebral congestion, is the extract of Belladonna. He gives it in the form of pill ; beginning with about one or two grains *per diem* at first, and gradually raising the dose to four or five grains, provided no affection of the sight or any other intoxicating symptom is induced. In some cases, he conjoins with advantage the use of a decoction or infusion of Valerian. But neither this latter remedy, nor yet the oxyde of Zinc, nor the nitrate of Silver—although all of them have been found occasionally useful—can be trusted to alone. In general, the more frequent the paroxysms are, the more hopeful we may be of making an impression on the disease: it is when two or more months intervene between each attack, that this Neurosis is usually most obstinate and intractable. In such cases, the Belladonna should be administered for a week or two before the expected invasion. When this is preceded by a distinct *aura Epileptica*, a strong dose of Ammonia will sometimes serve to ward off the attack: the patient therefore will do well to carry a small phial of the volatile alkali in his pocket. In some cases, the paroxysms of Epilepsy may be arrested for several months by the use of the Belladonna ; but nevertheless they ultimately return almost as frequently as ever, in spite of the prolonged continuance of the remedy. It is under such circumstances as these that the decoction of Valerian root, or of Orange leaves, should be exhibited at the same time.

Dr. *Debreyne* does not conceal the fact that several writers have recorded their opinion that his favourite remedy has utterly failed in their practice. He mentions particularly a report by M. *Picard* of 22 cases that were treated with it by M. *Ferrus,* in the Bicetre Hospital, in 1837. He attributes its failure in these cases—in part at least—to the injudicious manner in which the extract was given ; the doses being far too large, and carried to such an extent as to prove rather poisonous than sanative. This is certainly not the way to give a fair trial to the remedy.

Our author remarks that, ‘ if in symptomatic Epilepsy, after the removal of the exciting cause, the paroxysms continue from a sort of nervous habitude, they will be best obviated by the Belladonna ; and, in the event of this failing, by the use of Quinine and Valerian.’

*Hysteria.*—The following formula is very highly lauded by Dr. D. in the treatment of this too common disorder.

℞ P. Camphoræ . . . ℥ss.

P. Assafœtidæ . . . ℥ss.

Extr. Belladonnæ . . ℈iv.

Extr. aquos. Opii . . ℈j.

Mix and divide into 120 pills ; commence with two at first *per diem,* and gradually increase the dose to six in the 24 hours ; they should always be taken before food. Occasionally a wine glassful of the infusion of Valerian or Orange leaves may be given with much advantage along with each dose of the pills.

Dr. D. is in the habit of administering them also for the cure of general or partial nervous Trembling, and of *Chorea.* Sometimes he exhibits, in the latter disease, the Belladonna by itself; and, he says, very generally with success. When it fails, he has recourse to cold bathing. No allusion is made to the use of Steel in the treatment of this complaint by our author ; an omission that seems the more strange, as we shall afterwards find that he is so partial to ferruginous medicines in the treatment of many diseases of debility. According to our opinion, *the* remedy for Chorea is the carbonate or sesqui-oxyde of Iron, especially when administered in any bitter infusion.

*Neuralgia.*—“ For the last fifteen years, we have been in the habit of using with the greatest success, in all the forms of neuralgia,—Sciatica excepted—the Belladonna as an external application. Our favourite formula is this :—

℞. Extr. Belladonnæ . . ℥ss.

Opii pulveriz. . . . ℈ij.

Adipis suis . . . ℥ss.

Olei thymi . . . . ♏vj. M.”

A portion of this ointment, as big as a hazel-nut, is to be well rubbed upon the affected part two or three times a day, or whenever the paroxysms of pain are severe. The rubbing should be continued for eight or ten minutes at a time, until the ointment is quite absorbed by the skin: a little saliva may be added every now and then to promote the absorption. Let it be remembered that the use of this ointment should be at once suspended, if the sight becomes very sensibly affected, or any unpleasant cephalic symptoms supervene. In very obstinate cases, Dr. D. conjoins the internal administration of the extract of Belladonna or Opium with the use of the above pommade; but in the majority of instances, this is unnecessary, as the pain will very generally yield to the outward application. We employ it, he says, specially against facial neuralgias and other local painful affections of a nervous character, the Megrim, &c. In one very severe case of Neuralgia, which had lasted for nearly twenty years, and which had resisted our author's quasi-specific pommade, as well as a score or two of other approved remedies, the pain, which was seated in the skin, over the lower left ribs, at length yielded to the application of the Vienna Caustic paste, so as to produce a pretty large eschar upon the affected part. With respect to the treatment of Sciatica—which, as we have seen, Dr. D. separates, in a therapeutic point of view, from the other forms of Neuralgia —his usual plan is first of all to order the application of several *volant* blisters along the course of the affected nerve; and, if these do not quickly succeed in relieving the pain, to have recourse to his terebinthinate mixture, which is only a modification of that recommended first by Professor *Recamier*, and subsequently by Dr. *Martinet*. The formula is this:

| | | |
|---|---|---|
| ℞. | Aquæ lactucæ . . . . | ℥viij. |
| | Olei volat. terebinth. . . | ℥j. |
| | Gummi Arabic . . . . | ʒv. |
| | Syrupi simpl. . . . . | ℥iiss. M. |

The dose, a large table-spoonful, in a glassful of rice-water, three times a day, upon an empty stomach. Dr. *Debreyne* recommends at the same time the external application of an embrocation—composed of Spirits of Turpentine, Ammonia, Camphorated Spirits of Wine, and melted lard,—with which the affected parts are to be vigorously rubbed night and morning. In still more intractable cases, he has recourse to the use of moxas, applied over the seat of the chief pain; the best point generally for their application is immediately behind the great trochanter. In conclusion, he frankly admits that the use of his favourite Belladonna ointment is seldom efficacious for the relief of Sciatica.

*Paraplegia and Local Palsy.*—" Before we were acquainted," says Dr. *Debreyne*, " with the special action of Nux Vomica on the spinal-marrow, we were in the habit of trusting almost entirely to the use of moxas, applied over the lumbar or sacral vertebræ, for the cure of Paraplegia. But, for the last twenty years, we have invariably commenced our treatment of this disease with the alcoholic extract of the Nux Vomica, exhibited in the form of pills, each containing one grain of the extract." He begins with one, and gradually increases the dose until six—two at three different times—be taken in the course of the twenty-four hours. Whenever the patient experiences cramps and spasmodic twitches or tetaniform rigidity in the limbs, the action of the medicine must be narrowly watched; and it will be prudent either to diminish the dose, or even to suspend its use altogether, if these symptoms become excessive. The object should be to keep up the nervous excitement in a moderate and safe degree for a considerable space of time. If after a month or two's use of the Vomica, no decided benefit is obtained, Dr. D. advises the application of one or more moxas over the lumbar region.

He very properly cautions his readers not to expect the same benefit from the use of the Nux Vomica in the Hemiplegic, as in the Paraplegic, forms of Palsy. It may, indeed, prove serviceable in some cases of the latter, where there is every reason to suppose that the sanguineous coagulum within the cerebral substance has been nearly or altogether absorbed; but in no case of this description should we be sanguine of doing much good.

For the cure of *Amaurosis*, our author relies chiefly on the repeated application of small blisters in the neighbourhood of the affected eye, first on the temple and then over the eyebrow. In obstinate cases, the blistered surface should be sprinkled with a powder composed of starch and strychnine—about a fifth of a grain may be used at first, to be gradually increased. When this treatment fails, a seton should be tried. Dr. D. has used with very decided success a collyrium, containing some extract of Belladonna, in a good many cases of day blindness or *Nyctalopia.*

He also mentions a simple remedy for nervous *Deafness*, which may deserve notice. Let the patient fill his mouth with the smoke of Tobacco, or of any other dry aromatic plant—Sage, for example—and then make a forced expiration, while the mouth and nostrils are closed: this should be done several times in the course of the day. The smoke enters the Eustachian tube, and thus produces a slight stimulant effect upon the internal ear. The remedy can do no harm; and this is saying a good deal in its favour, considering the nature of many of the means of acoustic medication. It is best suited to those cases where the deafness has supervened on some catarrhal complaint, and whenever we have reason to believe that the pharyngeal end of the Eustachian tube has become thickened or obstructed.

*Asthma.*—" For the last twenty-five years we have seldom prescribed any other formula but the following:—

| | | |
|---|---|---|
| ℞. | P. Inulæ Elecamp. . . . | ℥ss. |
| | Flor. Sulphuris . . . . | ℥ss. |
| | P. rad. Belladonnæ . . . | ℈iv. |
| | P. rad. Scillæ . . . . | ʒj. |
| | Kermes min. . . . . . | ℈j. M. |

To be divided into 90 powders, of which one is to be taken three times a day."

Our author assures us that he has witnessed excellent effects from this remedy, not only in asthma, but also in a variety of chronic pectoral affections, when they are unaccompanied with fever or inflammatory irritation; as, for example, in what has been called Catarrhal Phthisis, and so forth. To allay the cough in such complaints, he combines the use of the Iceland moss jelly with the anti-asthmatic powders. When these fail—which, according to his report, is not often the case—he advises a trial of the Stramonium inhalation, and also of a strong infusion of the *Camphrée* of Montpelier (Camphorasma Monspeliaca)—with the medicinal virtues of which our author was first made acquainted by a writer in the Revue Medicale for March 1821. During the paroxysms of asthmatic dyspnœa, he recommends a mixture containing the extract of Belladonna, Oxymel of Squills, Kermes Mineral and Orange-flower Water.

In *Hooping-cough* also he again mainly trusts to the internal use of the Belladonna, in the form of its powdered root. This remedy was employed with very marked success by *Wetzler* during a severe epidemic of this disease that prevailed at Augsbourg in 1810; and it was about seven years afterwards that our author first gave an extensive trial to it. The dose of the powder must, as a matter of course, depend upon the age of the child, its constitution, the character of the existing symptoms, and so forth; but, if we state that a third of a grain should be given to a child twelve months old, twice or thrice a day, it will not be difficult to apportion the doses to other ages. When the fits of coughing are usually followed by vomiting, the powder should be given very soon after this has ceased. We need scarcely say that, if symptoms of inflammatory irritation be present, these must be subdued by the appropriate remedies, before recourse is had to the use of the Belladonna powder.

In accounting for the failure of his favourite remedy in the hands of several medical men, who have recently published the results of their experience with it, Dr. D. alludes, with much judgment, to some of those causes or influences which should always be attended to in estimating the virtues of a medicine in any epidemic disease, and the neglect of which, in the present day, has induced such striking discrepancy of opinion on various practical points among different writers, as is anything but creditable to the sagacity of professional men.

"Before," says he, "any one can fairly and satisfactorily determine the medicative virtues of Belladonna, or indeed of any other remedy, in Hooping-cough, by the effects which it may produce in any particular epidemic, it is absolutely necessary that he should imitate the example of such observers as *Sydenham* and *Stoll*, and have first carefully noted the type and genus of the epidemic itself, in order that he may know *in limine* whether it be inflammatory, or catarrhal or bilious, &c. in its nature. He should moreover have attentively ascertained the character not only of the medical constitution of the season, but also of the prevailing diseases of the preceding as well as of the current year, so that he may be able to determine, if possible, their cor-relations and mutual dependencies. If the existing epidemic proves to have an inflammatory character, it is scarcely necessary to say that the use of antiphlogistic measures is an indispensable preliminary in the treatment; whereas, if it has a bilious type, we must trust more to the use of emetics and purgatives, before having recourse to the exhibition of the Belladonna."

Before dismissing the subject of Coughs, we may state that Dr. *Debreyne* very strongly recommends the internal use of the extract of Belladonna, in the form of mixture, in most coughs of a nervous nature occurring in adults. He mentions the case of a woman, who had been afflicted with a violent convulsive cough for upwards of twelve years, that was speedily relieved by this remedy—dose, one grain two or three times a day. It is equally serviceable in the cure of obstinate Hiccup, and of any spasmodic constriction of the throat and larynx.

An ointment, composed of four parts of the extract and twelve or fifteen of spermaceti ointment, may be most advantageously used with much benefit in many cases of contraction of the anus, and painful affections of the cervix uteri; also in various neuralgic complaints of the bladder and urethra.

We now proceed to notice some of the most common gastric and intestinal affections, for the purpose of explaining our author's therapeutic views; and first of all we take the subject of—

*Vomiting.*—In the vomiting that may be considered to be nervous or spasmodic in its nature—*i. e.* when it is not connected either with inflammation or any bilious disturbance of the stomach—he recommends very highly the use of Colomba powder: it possesses, he says, a sort of specific virtue in such cases nearly as great as Bark does in Agues. He gives it in doses of from 15 to 20 grains in two or three spoonsful of red (French) wine, before meals. The addition of a few grains of magnesia, or of a minute dose of opium, may be necessary, if much acidity or gastralgia be present; and, should the patient be feeble and anæmic, the subcarbonate of iron may be very advantageously combined with it. Opium is freely used by Dr. D. in various abdominal affections, after the state of the intestinal secretions has been ascertained to be tolerably healthy. The following quotation will shew how highly he rates its value.

"We treat all internal pains whatsoever, and more especially those of the abdomen, with some preparation of opium—provided always they are not connected either with acute fever, or with inflammation, or gout. We may, indeed, make a still more general assertion, and say that it is to the use of opium—which is *the* antidote of pain—that we mainly trust for the relief of all painful chronic diseases. If, along with the element of pain, there should happen to be co-existing a rheumatic principle—whether this shew itself externally or in some internal organ—we associate the use of rubefacients and other appropriate medicines along with that of opium. Without this most valuable drug, there could be no possible medication for a multitude of chronic diseases. If we were deprived of it, we should ourselves instantly abandon the practice of the healing art. *Sydenham* thanked God for His gift of opium to mankind for the cure of so many of the ills to which we are liable; and we can safely affirm, as far as relates to our own practice, that never a day passes over that we have not occasion to exhibit opium in some form or another. How admirably it acts, almost as a specific, in most cases of Dysentery, not to enumerate a host of other maladies."

*Phthisis.*—Although Dr. D. classes this too-frequent scourge of France among the chronic Phlegmasiæ, he expressly says that he does not regard it as at all of an inflammatory character in its early stages. During the course of the disease, there is a strong tendency, as every one knows, to the frequent occurrence of a pneumonic and pleuritic attack; but this is to be regarded only as an *epi-phenomenon,* and not as a necessary symptom. With respect to Treatment, we find that our author has almost entirely renounced—after the experience of their utter inefficacy—the employment of nearly all remedies, save and except the insertion of a seton in some part of the chest, and the persevering use of Iceland-moss jelly in large quantities, not forgetting the Opium or Belladonna once or twice in the course of the 24 hours. He recommends the same line of treatment in those cases of chronic purulent Catarrh, the symptoms of which so closely resemble those of genuine tubercular Phthisis; and, in not a few instances, has a cure taken place under their use, when the case had seemed to be utterly hopeless.

From the Chest we pass on to the Stomach. After delivering some excellent remarks on the mode of distinguishing *gastralgic* from *gastritic* pain, our author exposes, with no less truth than severity, the melancholy mistakes that have been committed of late years by so many of his countrymen, since the prevalence of the Broussaian doctrines. He shews that the existence of an inflammatory state of the stomach may generally be diagnosticated by observing the effects which different kinds of food have upon the gastric pain. If, for example, farinaceous and milky substances can be taken well, while those of an animal nature give rise to a sense of uneasiness, we may very generally presume that there is a greater or less degree of actual gastritis. If the reverse be the case, and if light animal food, such as chicken-tea or mutton-broth, be borne best, we may feel assured that there is no inflammation, however troublesome the gastric uneasiness may be. The diet may therefore be regarded as a most useful *exploratory* means of diagnosis.

" It often happens that the epigastric pain does not yield to leechings and low diet; and woe be to the physician who pertinaciously seeks to combat it by continuing the use of the same means, and who has not learned to modify his treatment according to the varying condition of each case. For the relief of the gastric pain, which resists the application of leeches, &c., opium is often an excellent remedy: a light preparation should always be preferred, and it will be well to exhibit it in some mucilaginous vehicle.* If, however, this does not succeed in the course of a day or two, we should then have recourse to a volant blister. Should this also fail, we shall have good reason to believe that the epigastric uneasiness is more or less dependent upon an atonic state of the stomach; and this we can generally determine by having recourse to the *explorative* diet of which we have spoken. Should such be found to be the case, we must allow the patient more nourishing food, and we should try the effect of an infusion of Rhubarb or Colomba-root, to which may be added a weak opiate, if deemed necessary."

In *Chronic Hepatitis*, our author strongly recommends the use of emollient poultices on the hypochondriac region, applied every night, and also during the day, if the patient keeps his bed. They produce a local diaphoresis, which is often very serviceable in relieving the internal congestion. The occasional use of a tepid bath at the same time will much promote the cure; for the skin is generally very dry and lichenous in chronic liver complaints. Emollient aperient enemata are also very useful. Saline purgatives, dissolved in a large quantity of herb-tea, to be followed by repeated doses of rhubarb—which has long had the reputation

---

* A favourite formula of our author is this:—

℞. Aquæ lactucæ . . .. .. ℥viij.

Laudani Sydenhami. . .. ♏l.

Gum. arabic. .. .. .. ʒvj.

Syrupi simpl. .. .. .. ℥ij.

Bicarbonat. sodæ .. .. ℈ij. M.

A table-spoonful or two to be taken twice or thrice a day.

of directly promoting the flow of the bile—are always more or less necessary.

In various chronic hepatic affections and visceral obstructions, the following formula has been found by Dr. D. to answer exceedingly well.

℞. Pul. Aloes . . . . . . ʒij.

Sapon Hispanic.

Pulv. Rhei.

Ferri Subcarbonat. . āā ʒiv.

Potassii Ioduretti . . . ʒij. In pil. 120 divide.

Dose—From two to six pills in the course of the day.

If these pills do not prove to be sufficiently purgative, the patient should be instructed to drink some aperient mineral water to aid their action.

*Erysipelas.*—" The treatment which we usually adopt in recent cases of this disease, before the formation of pus has taken place, is the abortive and empiric plan followed by *Dupuytren,* and which he derived from the practice of M. *Petit* of Lyons. This method consists in applying a volant blister to the very centre of the inflamed part. The object, which we have in view by this bold and seemingly not very rational mode of treatment, is to arrest the internal inflammatory action, and to cause it, so to speak, to *abort,* by drawing to one circumscribed spot of the skin all the violence and *raptus* of the existing disease. It is quite true that this powerful concentration and sudden localisation of the inflammatory action may, in consequence of being excessive, induce gangrene of the blistered part; but this accident is of very rare occurrence indeed. Out of between 30 and 40 cases treated by us, we have only met with a single instance of it; and this occurred in a cachectic patient, whose constitution was altogether unhealthy. The application of a blister to the knee was, in this case, followed by the formation of a gangrenous eschar in the part, and the eventual consequences were extensive detachment of the surrounding skin, and considerable suppuration. Of late years, however, it would seem that this unpleasant result of the vesicatory treatment has been observed several times in the Hôtel Dieu, at Paris, and that antiphlogistic measures have been on the whole very successful; whereas, during the years 1813, 1814, and 1815, it was remarked, by all who followed *Dupuytren's* practice, that bleeding, &c. produced but little benefit, while blisters seemed to be quite a specific remedy."

*Chronic Cutaneous Diseases.*—Our author, without troubling himself with the divisions and subdivisions of these diseases adopted by most dermatological writers, groupes them together under the general appellation of *dartres,* and lays down some general therapeutic directions that may be applicable to all. A mild unirritating diet, more vegetable and milky than animal, emollient refreshing drinks—one of the best of which is whey—and the more or less frequent use of warm-baths, should never be omitted. Dr. D. throws overboard, as being utterly useless, the farrago of what have been called *depurative* remedies, such as the infusion of Fumaria, Dulcamara, Bardanum, Saponaria, Scabiosa, &c. &c. and he supports his own opinion on this point by quoting that of *Alibert.* The only internal remedy that he uses is the following:

℞. Flor. Sulphuris . . . . . . ʒiv.

Sulphureti Antimon. rubri . . ʒj.

Calomelanos . . . . . . . . gr. xij. M.

To be divided into 40 powders, of which one is to be taken twice a day.

The external treatment is, according to his experience, of much greater importance than any internal remedy. As a matter of course, as long as there is any irritation present, the more simple the baths and other external applications are, the better; but when this has been subdued, we should as early as possible have recourse to such as are slightly stimulant and exciting. Of these a sulphur bath—prepared by adding five or six ounces of sulphuret of potash to an ordinary bath—will be found very convenient. The use of this should be continued steadily for two or three months at least. If the *dartrous* affection is limited to the legs, as is often the case, it may be sufficient to use a partial sulphur bath to them alone: from one to two ounces of the sulphuret may be added to the requisite quantity of water. This is a far better application than all the ointments and lotions that are so generally in use. "If the local atonic *dartres* prove very obstinate and will not yield to sulphuretted baths and lotions, we are in the habit of trying an ointment—composed of ten drachms of the sulphuret of potash to six or eight ounces of lard, flavoured with oil of thyme—and usually with good effect: the strength of the ointment must be varied according to the degree of irritability in the affected parts." Dr. *Debreyne* tells us that, for the last five and twenty years, he has made use of no other application than the sulphuretted ointment—weak or strong, according to circumstances—in the numerous varieties of *Tinea,* or Scalled-head.

Of the Asthenic class or division of chronic diseases, none is of such frequent occurrence, and therefore so important in the eyes of the practical physician, as atonic Dyspepsia, or, as our author designates it, *Gastro-atony.* The following remarks are picked out from the description which he gives of it.

This disease is of very frequent occurrence, especially among women who are subject to leucorrhœal and chlorotic affections. Its most obvious symptoms are loss of appetite, uneasiness and sense of distention after eating, flatulence and often nausea and sickness, a feeling of sinking weakness and craving, more rarely of dragging, pain about the stomach; constipation; tongue white; taste more or less depraved, but without being bitter or clammy, as in bilious derangements; loss of muscular strength; tendency to nervous ailments, and these usually accompanied with great irritability of temper. Very generally (and this remark, by the bye, it is especially important to attend to) an invigorating animal diet agrees better than a farinaceous and vegetable one—a circumstance that is very significant, and obviously excludes the idea of any phlogistic or irritative element being present. The mere circumstance of there being some degree of pain in the epigastrium, even although this be increased on pressure, is by no means a sufficient reason for suspecting the existence of any inflammatory action; for this pain may be truly called an *atonic* pain, and can be relieved neither by opiates nor antiphlogistics, but only by appropriate tonics.

With respect to the *treatment* of Gastro-atony, the mere regulation of the patient's diet will often suffice to relieve the milder and less chronic forms of it. He should avoid the use of much vegetable or farinaceous food, and live chiefly on animal meat and good bread, with or without an allowance of a light sound wine, according to circumstances. The drinking of large quantities of hot drinks, such as tea, coffee, &c. is most injurious. Our author very pithily remarks, ' pour les maladies chroniques apyretiques, medicaments secs; et pour les affections aiguës, medicaments liquides :' the remark would apply better to *food* than to *medicines*. Steel and vegetable bitters are by far the best remedies that we can administer, more especially when there is any leucorrhœal or chlorotic ailment. Rhubarb is the preferable aperient medicine in such cases. If the patient be subject, as occasionally happens, to attacks of nervous or spasmodic vomiting, Dr. D. always has recourse to the Colomba powder. If much pain accompany this unpleasant symptom, a small portion of opium should be added to the Colomba. Ice too will often be both grateful and useful, not only in allaying the irritability of the stomach, but also in giving it tone.

When *Leucorrhœa* accompanies this atonic state of the digestive organs, Dr. *Debreyne* recommends the use of the following pills :—

℞. Ferri Subcarbonat. . . . . ʒiv.

P. Cassuvii (Cashew) . . . ʒiv.

P. Aloes . . . . . . . . ʒj.

Terebinth. Veneti . . . . . . q. s. ut fiat massa in pil. cxx. div.

Dose: one or two to be taken, along with some bitter tincture or infusion, twice or thrice a day.

A somewhat similar formula will be found very efficacious in most cases of *Chlorosis*.

*Dropsy*.—The following extract gives a good summary of our author's views respecting the treatment of this disease, when it is not dependent upon any organic visceral lesion.

"We should always make sure of one of the outlets, by which nature usually seeks to evacuate the serosities effused within the splanchnic cavities. Now, as in the cure of Dropsies, the serous evacuations most frequently take place by the bowels and kidneys, it will be prudent to act upon both of these emunctories, by combining the use of diuretics with that of hydragogue purgatives. At the same time, we should prescribe a dry and tonic diet, consisting chiefly of broiled or roasted meats, bread, and a certain allowance of light wine. The patient should be directed to take as little fluid food or medicine as possible, and he should therefore seek to quench his thirst with fruit, ice, and such like things."

The favourite medicine of Dr. D. in dropsy is a medicated wine, composed of

Rad. Jalapæ contus. . . . ʒiiss.

—— Scillæ contus. . . . . ʒiiss.

Pot. Nitratis . . . . . . . ʒv.

Vini Albi . . . . . . . . ℔j.

Dose: from one to three tablespoonfuls thrice daily.

The number of alvine evacuations need not exceed six or eight in the 24 hours. The remedy acts in some cases chiefly on the bowels, in others chiefly on the kidneys, while in a third set of cases both emunctories are powerfully affected at the same time. When patients object to the use of this wine, or when it appears to disagree with the stomach, we may have recourse to the use of the following pills :—

℞. Pulv. Digitalis . . . . ℥iv.

Pulv. Scammoniæ . . . ℥ij.

Pulv. Scillæ . . . . . . ℥ij.

Extract. Juniperi . . . q. s. Ut fiat massa in pil. cxx. dividenda.

Dose: from one to two pills three times a day, washing them down with three or four spoonsful of white wine, in a bottle of which half an ounce of nitrate of potash has been dissolved.

Dr. D. says that he has found these pills especially serviceable in cases of Hydrothorax and Hydropericardium. He is too experienced and candid a practitioner not to admit that we can seldom, or never, hope to effect a *permanent* cure in such cases; still it is an important thing to relieve our patients for a time, and prolong, if it be not given us to save, life.

In cases of Organic Diseases of the Heart, our author mainly relies on the internal administration of the nitrate in combination with tincture of Digitalis—given in much larger doses than are usually recommended—and on the application of leeches or blisters over the *cardiac region.* He seldom varies his plan of treatment, and assures us that, with these simple means, followed out for a due length of time, he has succeeded in relieving a great many patients, who had derived no benefit from a variety of other remedies.

In the closing Chapter of his Work, Dr. D. lays down some excellent general rules for the treatment of disease in the members, male and female, of those austere religious orders, who insist so rigidly on the observance of numerous and prolonged fasts, in addition to other modes of penance. Of these orders, the most conspicuous are the Chartreux and the Trappists, " whose establishments," we are told, " are now so numerous, and whose moral and religious influence, along with the benefits of agricultural improvement which they have introduced, are every day more and more felt and appreciated in many of the finest districts of France." A vast deal of harm has been done for many years past by the too common adoption of Broussaian principles by the medical practitioners of the Provinces; but now, thank God, the errors of Physiologism are most fully acknowledged and repudiated. As a general truth it may be asserted that the diseases of austere religionists will not bear well much depletion.

Even in acute disorders, the lancet should be sparingly used; and, instead of repeated bleedings, recourse should be had to the internal use of antimonials, and to blisters, &c. In fevers, when there is no distinct inflammatory localization, general bleeding should very rarely, if ever, be practised. Of chronic diseases, by far the most common among the Monastic Community are Gastro-atony and its usual concomitants of Dyspepsia, Colic, Gastralgia, general weakness, and so forth.

As for *Gastritis,* the term might be erased from the peculiar nosology to which we are at present alluding. Opium, either alone or in conjunction with other remedies, according to circumstances, is an admirable remedy in a vast number of the gastric and enteric disorders to which the Trappist brethren are liable. It would seem, from the statements of our author, that these monks are singularly exempt from the epidemics which prevail in the neighbourhood of their establishments. Even the cholera, in 1832, did not enter one of them throughout the whole of France.

This exemption he attributes to the temperance of their diet, and the calm unruffled tenour of their lives. He paints in glowing terms the joys of the peaceful life of the pious Cenobites.

"How greatly mistaken," exclaims our worthy author, "are they who suppose that religious penitents are gloomy, melancholy and hard-hearted men, or that they become the early prey of a tedious and painful death! No; their life is one long and blessed repose; or rather, as the Prophet says, it is a river of peace which calmly bears them on to the everlasting rest of God. They seem to the eyes of the worldly, who are altogether absorbed with the frivolities of the passing scene, to languish and die; and yet they are full of health and life, for they taste a peace and happiness of mind which the world cannot know: *Visi sunt oculis insipientium mori; illi autem sunt in pace.*"

